# Learning from Octopuses: Cutting-Edge Developments and Future Directions

**DOI:** 10.3390/biomimetics10040224

**Published:** 2025-04-04

**Authors:** Jinjie Duan, Yuning Lei, Jie Fang, Qi Qi, Zhiming Zhan, Yuxiang Wu

**Affiliations:** 1 School of Optoelectronic Materials and Technology, Jianghan University, Wuhan 430056, China; djjstudy@sina.com (J.D.); 13638665980@163.com (Y.L.); jf32607@163.com (J.F.); qiqi07230324@163.com (Q.Q.); 2 Institute of Intelligent Sport and Proactive Health, Department of Health and Physical Education, Jianghan University, Wuhan 430056, China; 3 School of Artificial Intelligence, Jianghan University, Wuhan 430056, China

**Keywords:** octopus, sensor, actuator, processor

## Abstract

This paper reviews the research progress of bionic soft robot technology learned from octopuses. The number of related research papers increased from 760 in 2021 to 1170 in 2024 (Google Scholar query), with a growth rate of 53.95% in the past five years. These studies mainly explore how humans can learn from the physiological characteristics of octopuses for sensor design, actuator development, processor architecture optimization, and intelligent optimization algorithms. The tentacle structure and nervous system of octopus have high flexibility and distributed control capabilities, which is an important reference for the design of soft robots. In terms of sensor technology, flexible strain sensors and suction cup sensors inspired by octopuses achieve accurate environmental perception and interaction. Actuator design uses octopus muscle fibers and movement patterns to develop various driving methods, including pneumatic, hydraulic and electric systems, which greatly improves the robot’s motion performance. In addition, the distributed nervous system of octopuses inspires multi-processor architecture and intelligent optimization algorithms. This paper also introduces the concept of expected functional safety for the first time to explore the safe design of soft robots in failure or unknown situations. Currently, there are more and more bionic soft robot technologies that draw on octopuses, and their application areas are constantly expanding. In the future, with further research on the physiological characteristics of octopuses and the integration of artificial intelligence and materials science, octopus soft robots are expected to show greater potential in adapting to complex environments, human–computer interaction, and medical applications.

## 1. Introduction

In recent years, the field of robotics has undergone a revolutionary transformation, with soft robotics emerging as a pioneering technology. Soft robots demonstrate remarkable potential across a variety of applications due to their unique characteristics, including flexibility and adaptability. Their durability, deformability, and ability to perform complex tasks in unstructured environments have attracted considerable attention from researchers [1,2,3].

Soft robotics is an interdisciplinary field that integrates concepts from biology, engineering, materials science, and computer science. By examining natural organisms—particularly soft-bodied creatures, such as octopuses, worms, and jellyfish—scientists have drawn significant inspiration for innovative robot designs [4,5]. These organisms exhibit exceptional movement, manipulation, and adaptability, providing a wealth of ideas for the development of advanced soft robotic systems [6,7].

Marine animals have evolved to thrive in fluid environments, developing highly adaptable movement and body structures over time [8]. The wave-like swimming of fish, the flexible tentacle movements of octopuses, and the pulsatile propulsion of jellyfish are all remarkable examples of nature’s ingenuity in achieving efficient locomotion within complex environments [9]. The octopus, a quintessential invertebrate, is particularly notable for its highly flexible tentacles that possess multi-degree-of-freedom movement capabilities. Composed of both muscular and gelatinous tissues, the tentacles of octopuses can perform a range of complex actions, including grasping, coiling, and propulsion. This unique structure and functionality serve as valuable inspiration for the design of soft robots [10,11].

Interestingly, the neural architecture of octopuses is decentralized; approximately 40% of their neurons reside in the brain, while the remaining 60% are distributed throughout their tentacles [12]. This distributed processing significantly enhances the octopus’s responsiveness and ability to execute multiple tasks simultaneously, offering insights for the development of distributed processing systems in robotics, as well as strategies for task allocation between primary and auxiliary processors [13,14,15]. The development of soft robots inspired by octopuses primarily manifests in the implementation of flexible strain sensors, the design of sensor arrays on suction cups, and the innovative creation of bionic sensor materials [16,17]. Soft robots can be classified based on their actuators’ power sources into three main categories: pneumatic actuators, electric actuators, and hydraulic actuators [18,19,20]. Additionally, control algorithms, such as proportional–integral-derivative (PID) and linear quadratic regulator (LQR), significantly enhance the motion accuracy of these actuators when integrated with traditional systems. Research into the neural network systems of octopuses has led to the development of distributed control methods, which improve the overall control efficiency of robotic systems. This work also provides crucial insights for the exploration of asynchronous parallel optimization algorithms [21,22,23]. The significance of these studies is particularly pronounced in the context of the era characterized by big data and advanced computational power.

**Figure 1:**
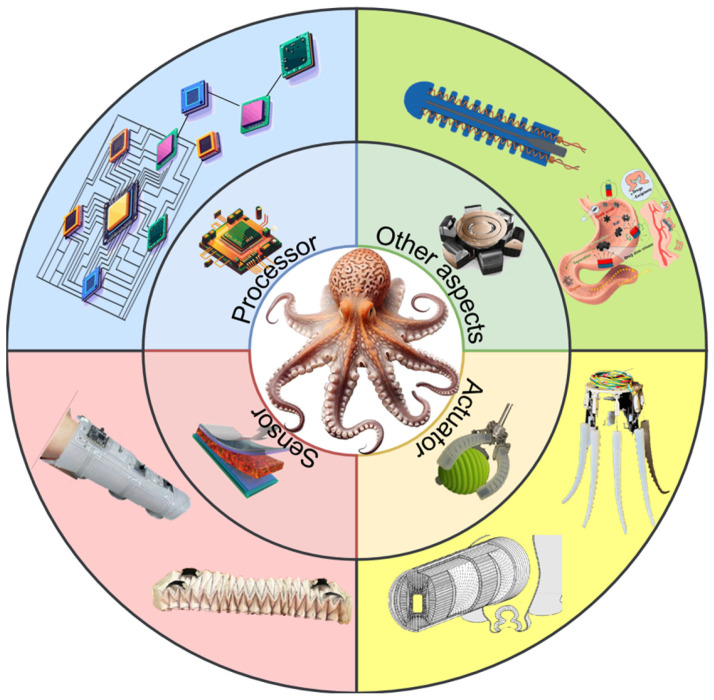
Schematic diagram of the relationship between the octopus’s body structure and the bionic soft robot.

In recent years, significant advancements have emerged from bionic research focused on octopuses, particularly in the areas of perception, control, actuator design, and processing systems. Further investigation into the physiological characteristics of octopuses holds substantial importance for the ongoing development of soft robots. This review systematically presents the bionic inspiration derived from octopuses and outlines the related research progress in soft robotics, specifically in the domains of sensing, actuation, and distributed processing (Figure 1). Furthermore, this paper introduces the concept of expected functional safety into the realm of soft robot research for the first time [24].

The primary function of sensors is to facilitate accurate data interaction between robots and their environments, while actuators are responsible for enabling movement and task execution. Processors play a crucial role in data processing and movement control [25]. Accordingly, this review begins with a succinct exploration of the physiological characteristics of octopuses, specifically examining the functions of their sensory organs, motor systems, and nervous systems. Subsequently, the discussion extends to the development of multi-processor systems modeled after the octopus nervous system. It also addresses advancements in flexible robotic sensing technologies and multi-sensor fusion algorithms inspired by the sensory organs of octopuses. Additionally, the evolution of various movement modalities and control technologies in flexible robots, influenced by the motor capabilities of octopuses, is introduced. The coordination between the main processor and co-processor is also examined.

Finally, the review concludes by outlining prospective future research directions in the field of bionics that can be applied to the development of flexible robots.

## 2. Octopus and Soft Robot Sensor Technology

Perception technology serves as the foundation for soft robots to achieve autonomous operation and intelligent behavior, and it is a crucial factor for their successful deployment in complex environments. The sensing technology of soft robots is central to their ability to perceive and interact with their surroundings. To enable these robots to respond intelligently to external stimuli, it is essential to integrate soft sensing components and flexible strain sensors into their structural design, allowing them to perceive a variety of environmental signals as feedback [26,27].

**Figure 2:**
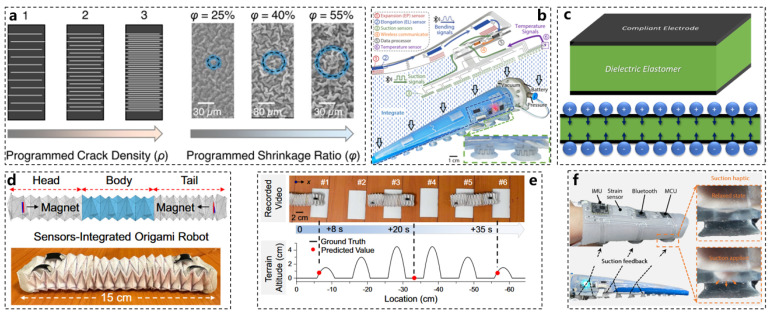
New sensor materials and their applications. (**a**) SWNT material characterization diagram, computationally guided PCAM sensor design during origami robot design, examples of different ρ and φ values. (**b**) E-SOAM integrates bending sensing (through strain sensors) and suction, embeds metal electronic components into the terminal holder, processes all signals at the terminal, and then transmits them wirelessly. (**c**) Schematic diagram of the charge distribution of piezoelectric materials that can be measured and grasped [32]. (**d**) Schematic diagram of the sensor-integrated origami robot. (**e**) ANN predicts the motion trajectory of the origami robot. (**f**) The three internal suction cups on the ventral inner surface of the E-SOAM haptic device transmit the sensory feedback of the end gripper suction to the human finger. The suction force applied to the finger to establish the tactile interaction between the end gripper and the finger is described in detail.

Currently, the availability of suitable strain sensors is vital for the perception and autonomy of soft robots; however, the variable shape and dynamic actuation of these robots present challenges for sensor manufacturing and long-term durability [6,7,28,29]. To address these challenges, a deterministic crack propagation mechanism has been proposed, characterized by a programmed crack array utilizing a micro-wrinkle strategy. A computational design of strain sensors has been developed to enhance both sensor modeling and stability. As illustrated in Figure 2a, environmentally stable single-walled carbon nanotubes (SWNTs) are employed to fabricate piezoresistive strain sensors. This design features a two-stage process that integrates the function of “programmed crack arrays in micro-wrinkles”, resulting in what is referred to as the PCAM sensor.

To validate the effectiveness of this mechanism, an origami robot was designed, as depicted in Figure 2d. Innovatively, this study also incorporated deep learning technology by employing an artificial neural network (ANN) to predict the motion trajectory of the origami robot based on data collected from the strain sensors [30,31]. The test results are presented in Figure 2e. This technology holds potential for applications in automated navigation and terrain mapping for robotic systems in future endeavors [26].

The perception of soft continuum robots presents a significant challenge in the field of robotics. The inherent high degree of freedom and redundancy in soft continuum structures complicates the simultaneous attainment of speed and accuracy in numerical approximate solutions to their nonlinear partial differential equation models [33,34]. Environmental perception in soft continuum robots faces several issues, including difficulties in aligning the sensor circuitry with the Young’s modulus of the robot’s body and challenges in designing a functional distribution of structures [5,26,35,36].

Observing how octopuses capture prey reveals an effective strategy: their slender tentacles utilize a unique mode of “bending wave transmission” to approach targets. This process begins with bending at the base, propagating along the arm toward the prey [37,38] Once a suction cup attaches to the target, the highly sensitive nerves within the tentacles and suckers quickly detect and secure the object. Inspired by this remarkable predatory behavior, the electronic integrated soft octopus arm (E-SOAM), illustrated in Figure 2b, is designed to enable extensive sensing and interaction capabilities. The E-SOAM features a wearable finger glove equipped with three internal suction cups on its ventral surface. When the suction cup attaches to an object, it generates a vacuum that pulls the skin of the glove, effectively translating the robot’s sensory feedback into a tangible human sensation. Consequently, the operator can discern the softness of the object through the tactile feedback provided by the terminal fixture and the wearable glove [39]. The E-SOAM offers a valuable design reference for enhancing the perception and interaction capabilities of soft continuum robots with their environments. By leveraging the bending transmission motion mode characteristic of octopus tentacles, along with wearable flexible tactile devices, we propose a sensor-integrated octopus arm robot prototype. This prototype successfully realizes an operation mode akin to that of octopuses, encompassing sensing, motion, and environmental interaction functionalities.

In terms of materials, the design of the bionic octopus sensor incorporates the functionality of a sensor into a conventional soft robot sucker, drawing inspiration from the octopus sucker. When a voltage is applied across the sucker material, the capacitance increases proportionally. Notably, when the sucker makes contact with an object, the capacitance experiences a significant increase, eliminating the need to lift the object to confirm a successful grasp [40,41,42]. The material structure of the sensor is illustrated in Figure 2c.

**Figure 3:**
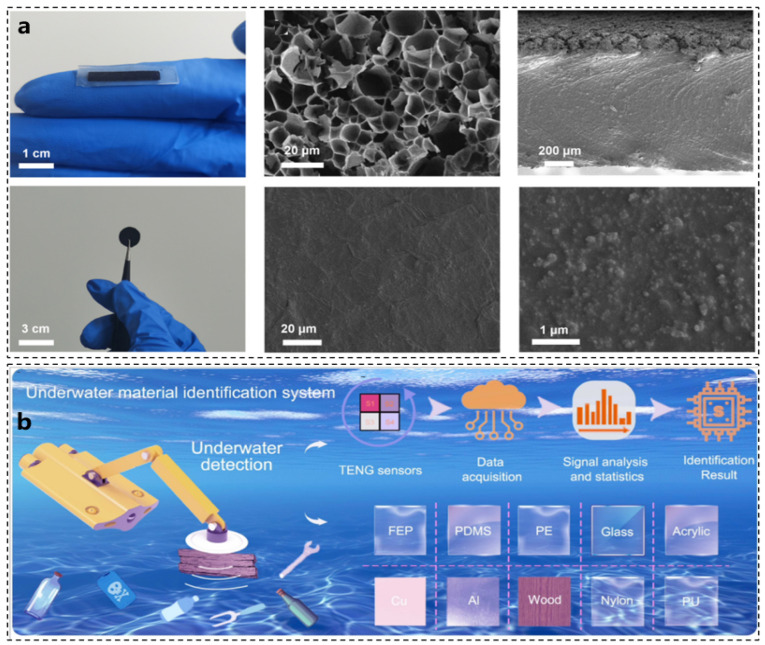
Sensor material design and detection performance optimization. (**a**) CNT/Ecoflex material diagram and SEM characterization [43]. (**b**) OI-TENG underwater material recognition system [46].

The bionic octopus sensor utilizes a CNT/Ecoflex conductive elastomer as its primary material, enabling the collection of the normal vector of the three-dimensional (3D) spatial force. Additionally, a laser-induced graphene sensing film is integrated into the Ecoflex to serve as the tactile wrist of the sensor, allowing for the discernment of both the magnitude and direction of the tangential component of the force. This integration endows the sensor with perceptual capabilities. Various shapes and scanning electron microscopy (SEM) images of the sensor are presented in Figure 3a [43].

Common sensors operating underwater face significant challenges in complex environments due to issues such as signal attenuation, biological fouling, and interference from water flow [44,45]. Drawing inspiration from the tactile system of the octopus sucker, a novel sucker tactile system has been developed that employs triboelectric tactile receptors to simulate the cephalopod-specific chemical sensor (CR) mechanism. The application of superhydrophobic treatment enhances the microstructure of the sensor’s surface, resulting in substantial improvements in output voltage, sensitivity, and response time. The constructed underwater material identification system (UMIS), based on the OI-TENG (Octopus-Inspired Triboelectric Nanogenerator), is illustrated in Figure 3b [46].

In addition to the continuous expansion of sensor types and technical applications, significant progress has been made in the development of sensor post-processing algorithms for soft robot perception technology. To achieve a comprehensive understanding of their environment, soft robots must integrate multiple sensors that can detect various parameters, including force, temperature, and shape [47]. This multimodal sensing capability enables soft robots to better comprehend and adapt to their operational environments [26,47,48]. Among the various techniques employed in multi-sensor fusion technology, the Kalman filter and its variants hold a prominent position. The Kalman filter, along with its extended versions—such as the extended Kalman filter (EKF) and the unscented Kalman filter (UKF)—are widely utilized for multi-sensor data fusion [49]. These methods are particularly effective in addressing sensor noise and uncertainty in both linear and nonlinear systems. In recent years, deep learning technologies, including convolutional neural networks (CNNs) and recurrent neural networks (RNNs), have gained traction in the realm of multi-sensor fusion. These advanced techniques can automatically extract and learn features from sensor data, enhancing the overall performance of perception systems [50,51,52].

The rapid development of multi-sensor fusion technology has made it a revolutionary enabler for soft robotic systems, especially for robust environmental interaction through adaptive sensor-actuator coupling. As shown in Table 1, a comparative analysis of Kalman filters, CNNs, and RNNs reveals the advantages, disadvantages, and application scenarios of several common sensor processing methods. This synergistic integration of model-based estimation and data-driven learning paradigms is driving the development of embodied intelligence in soft robots, enabling autonomous operation with sub-millimeter precision in unstructured environments.

## 3. Octopus and Soft Robotic Actuators

The actuator is a critical component of a soft robot, enabling movement and functionality. Table 2 shown the differences between octopus-inspired soft robot actuators and other biomimetic soft robot actuators. Given the inherent characteristics of soft robots, actuators must possess flexibility, deformability, and the ability to adapt to complex environments. Common modes of actuation in soft robots are primarily powered by various energy sources, including gas, liquid, electricity, heat, magnetism, and chemical reactions [6,53,54,55].

**Figure 4:**
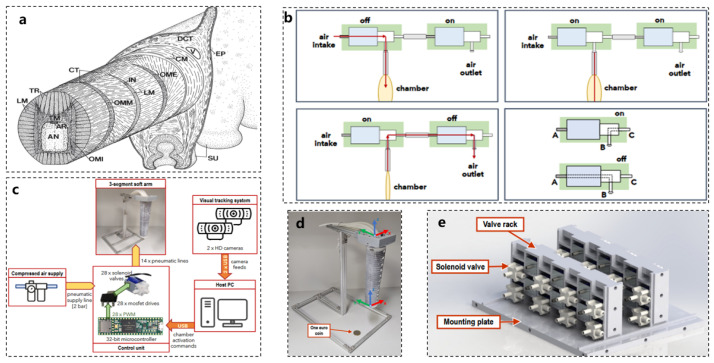
Pneumatic actuator working example reference [60]. (**a**) Octopus vulgaris arm muscle system [57]. (**b**) Pneumatic actuator control logic. (**c**) Diagram of the soft arm pneumatic actuation and control system, including an air source, 28 controllable solenoid valves, a 32-bit microcontroller, a visual tracking system, a host computer, and a three-segment octopus bionic soft arm. (**d**) An actual picture of the three-segment octopus bionic soft arm, comparing its size with a coin. (**e**) Pneumatic actuator control component solenoid valve.

The octopus serves as an ideal animal model for studying slender, flexible appendages. As illustrated in Figure 4a, the muscle system of the octopus arm consists of a dense three-dimensional array of muscle fibers and connective tissue, which extends along the entire length of the arm and encases the axial nerve cord [55,56,57]. Similar to the tentacles of the octopus, the movement of a soft robot is primarily facilitated by soft actuators. These actuators are crucial for enabling the movement of the soft robot, making their design and functionality vital to the overall composition of the robotic system. Depending on the specific application scenarios, the design, manufacturing, and control of soft actuators may vary significantly.

Pneumatic actuators are among the most prevalent types of actuators used in soft robots, utilizing compressed air to facilitate movement. By adjusting the air pressure and flow, pneumatic actuators can expand or contract, generating a range of movements. These actuators offer high flexibility and rapid response times, enabling complex deformation and movement patterns. They are widely employed in tasks such as grasping, walking, and crawling. However, the implementation of pneumatic actuators necessitates complex pneumatic systems and control devices, which may be constrained by the availability of air sources and the configuration of pipelines [20,58,59,60].

Inspired by the muscle structure of the octopus arm, researchers have designed a soft robotic arm constructed from soft silicone, as depicted in Figure 4d. This robotic arm comprises three slender segments arranged in series, with each segment containing multiple pneumatic chambers embedded within its silicone body. The solenoid valves illustrated in Figure 4e are employed to regulate the flow of compressed air into these pneumatic chambers. The control logic for the system is presented in Figure 4b. By selectively inflating the corresponding chambers, the arm can achieve bending, extension, and twisting movements, thereby facilitating the deformation of the current segment of the robotic arm. The overall control framework is depicted in Figure 4c.

By integrating segment deformation and simulating the function of the antagonistic muscles found in octopuses, this robotic arm can bend in multiple directions, adjust its length, and even twist around its central axis, thereby achieving movement capabilities akin to those of an octopus arm [60,61,62].

Hydraulic actuators utilize liquid pressure to facilitate movement and are particularly well-suited for applications that require substantial force. By controlling the flow of liquid through hydraulic pumps and valves, these actuators can generate significant thrust and torque, providing both high output force and precise control. They are ideal for tasks that demand high loads and meticulous regulation. However, hydraulic systems tend to be complex, incur high maintenance costs, and carry a risk of leakage [18,63,64].

**Figure 5:**
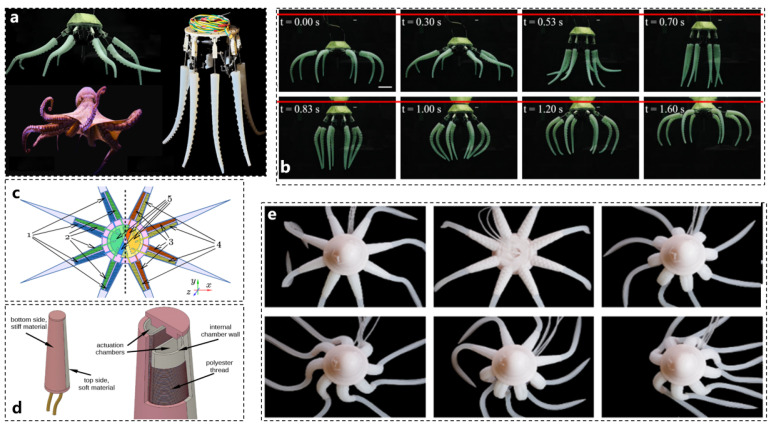
References to other power source actuator working examples. (**a**) Electric actuator complete machine example [71]. (**b**) Electric actuator motion posture [71]. (**c**) Hydraulic actuator modeling [65]. (**d**) Hydraulic actuator tentacle structure design [65]. (**e**) Hydraulic actuator motion posture [65].

Inspired by the morphology of octopus movement, novel actuators can be designed based on the principles of fluid drive. The actuator model developed for this purpose is illustrated in Figure 5c. This design employs silicone material for the drive chamber, with the driving fluid supplied to the robot through a flexible pipe that channels it to each arm via internal conduits within the robot’s body. As depicted in Figure 5d, the arm features a conical shape, and the drive chamber is constructed from silicone reinforced with multiple layers of polyester wire. The polyester wire is arranged in a spiral configuration along the conical clamping wall, ensuring that the actuator can bend and stretch effectively.

Testing has demonstrated that the bionic actuator shown in Figure 5e can achieve movement with minimal drive pressure when the drive is controllable. This actuator is capable of forward movement, rotation, and twisting around its main axis [60,65].

Electric actuators typically achieve motion tasks through direct motor control or methods such as electromagnetism and electrothermal effects. Among these, motor control provides high-precision angle and position regulation, enabling the actuator to perform complex grasping and manipulation tasks. The motor drive system is characterized by its rapid response, allowing for quick starts and stops. Additionally, motors exhibit high-energy conversion efficiency and can deliver substantial output torque within a compact volume. Their ease of integration with other sensors facilitates the creation of comprehensive intelligent control systems. Given the maturity of motor control technology, dynamic modeling is relatively straightforward, allowing for improved design of the entire system [66,67,68,69,70].

Figure 5a illustrates a novel bionic robotic swimming platform inspired by octopus locomotion. This platform achieves efficient swimming using only two standard motors. It combines asymmetric passive deformation arms with umbrella-shaped rapid return structures, effectively mimicking the arm movements and stroke timing of octopuses to enhance swimming efficiency. This actuator design significantly reduces the complexity of the drive system while maintaining excellent swimming performance, offering valuable insights for the development of low-cost underwater vehicles. The motion posture of the modified bionic soft robot is depicted in Figure 5b [71].

**Figure 6:**
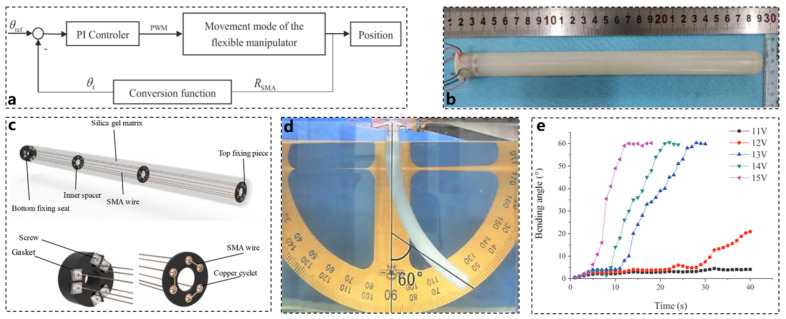
Actuator control algorithm and actuator materials [76]. (**a**) PI control logic suitable for SMA. (**b**) Actual picture of actuator designed with SMA. (**c**) Schematic diagram of actuator power based on SMA material. (**d**) SMA + PI control experimental device diagram. (**e**) Experimental data of SMA motion performance under different voltages.

In addition to the novel actuators previously discussed, the control algorithm governing the actuator is also of paramount importance. The flexible deformation of the robotic arm structure complicates accurate control. Figure 6b illustrates a proportional–integral (PI) control algorithm based on self-inductance resistance feedback from shape memory alloys (SMAs), which enhances the control accuracy of the bionic robotic arm. The soft robotic arm designed using SMA in conjunction with the PI control algorithm can be flexibly bent and precisely controlled. The overall system control block diagram is presented in Figure 6a.

In this design, SMA wire is selected as the actuator, while silicone material serves as the foundation for the flexible robotic arm, resulting in a bionic flexible arm. The SMA wire acts as an actuator that simulates the longitudinal muscle fibers found in octopus arms, embedded parallel to the central axis of the robot arm, as shown in Figure 6c. Three groups of SMA wires are symmetrically arranged around the axis of the flexible robot arm interface. By constructing a constitutive model for the SMA, the changes in parameters such as stress, strain, and martensite percentage due to temperature variations during the SMA phase transition can be effectively described.

The SMA is driven by pulse-width modulation (PWM) for electric heating. By adjusting the PWM duty cycle, the temperature of the SMA can be controlled, thereby regulating the movement of the flexible robotic arm. This logical relationship allows for the construction of a thermodynamic model for the SMA. Together, these two models elucidate the relationship between variations in the PWM pulse signal’s duty cycle and the resulting deformation of the SMA. It is well-known that flexible actuators exhibit significant inertia and hysteresis, leading to slow changes in motion error during operation. Consequently, the differentiating component of the PID algorithm is less effective. Therefore, a PI controller is employed to accurately manage the movement of the bionic flexible robot arm. Control angle measurements were conducted using the device depicted in Figure 6d. Experimental results confirmed that varying driving voltages produced different bending angles and response speeds of the robot arm. Furthermore, it was observed that higher voltages corresponded to faster angle changes, as illustrated in Figure 6e [72,73,74,75,76].

By studying the movement and associated organ characteristics of the octopus, we have significantly broadened our perspectives on actuator design. This represents a crucial milestone in the optimization of soft robot design and the development of enhanced functionalities.

## 4. Octopus and Processor Architecture and Intelligent Optimization Algorithms

**Figure 7:**
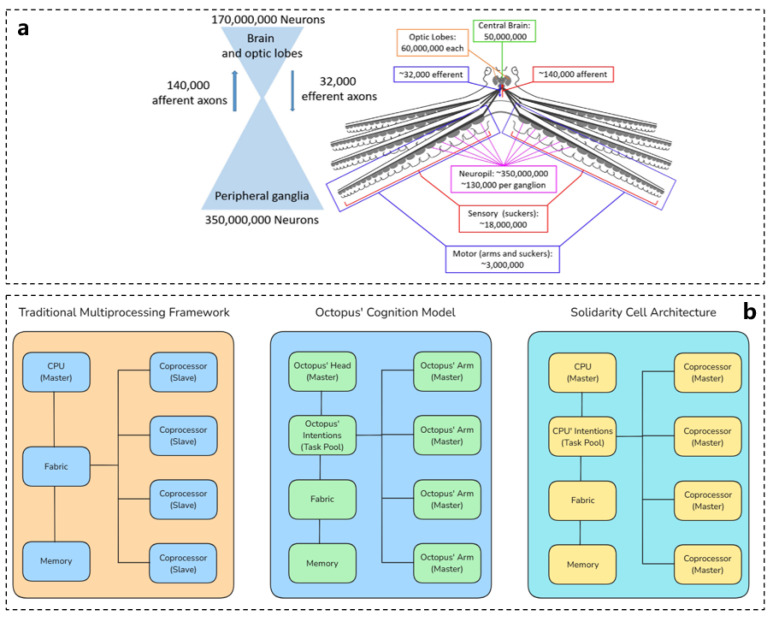
Octopus neural network explaining distributed processing. (**a**) Schematic diagram of octopus neural network and neuron distribution [83]. (**b**) Evolution diagram of distributed processing architecture inspired by octopus [82].

Octopuses are regarded as the most intelligent of all invertebrates. Research has shown that only 40% of the neurons in octopuses are located in the brain, while the remaining 60% are distributed throughout their eight tentacles. The distribution of these structural functions is illustrated in Figure 7a. A significant portion of the octopus’s nervous system resides within its arms. Each arm contains a nerve cord, known as the arm or axial nerve cord, which runs parallel to the suckers. Along the ventral side of each arm, 200 to 300 suckers are staggered, and the nerve cord comprises a dense, continuous network of neuropil that extends from the surrounding layer of monopolar neuronal cell bodies [77,78,79,80].

The nerve fibers at the base of each sucker are enlarged, forming structures commonly referred to as ganglia, while the segments between these ganglia are termed interganglionic regions. Notably, the octopus brain does not issue commands for every minor movement of the arms; instead, many decisions are made autonomously by the arms themselves. This distributed arrangement of neurons enables the octopus’s arms to solve problems independently. For instance, when an octopus explores a cave and discovers edible items, its arm can simultaneously open shellfish. This distributed control architecture facilitates effective and computationally efficient control of the arms [79,81].

The structure of the octopus’s nervous system and the manner in which the brain communicates with the arms can inform the design of system architectures. A multi-processor distributed processing system allocates control between a main processor and a coprocessor, allowing the coprocessor to share workload tasks and thereby reducing the burden on the main processor. This distributed control approach is often referred to as hybrid control, which describes a hierarchically organized system where a discrete control mode is selected at a higher level, while different continuous controllers operate at a lower level for each mode. The control strategy employed by octopuses exhibits several characteristics that align with hybrid control principles. The evolution of the distributed processing architecture inspired by the octopus nervous system is illustrated in Figure 7b [22,82,83,84,85].

In addition, an intelligent optimization algorithm inspired by the characteristics of the octopus neural network has been developed. The Octopus-Inspired Optimization Algorithm (OIO) is an advanced optimization technique that leverages the unique features of the octopus’s neural system. It employs a multi-level hierarchical strategy to achieve global search and local optimization through asynchronous parallelism. This effective combination of search methodologies significantly enhances both search efficiency and adaptability.

**Figure 8:**
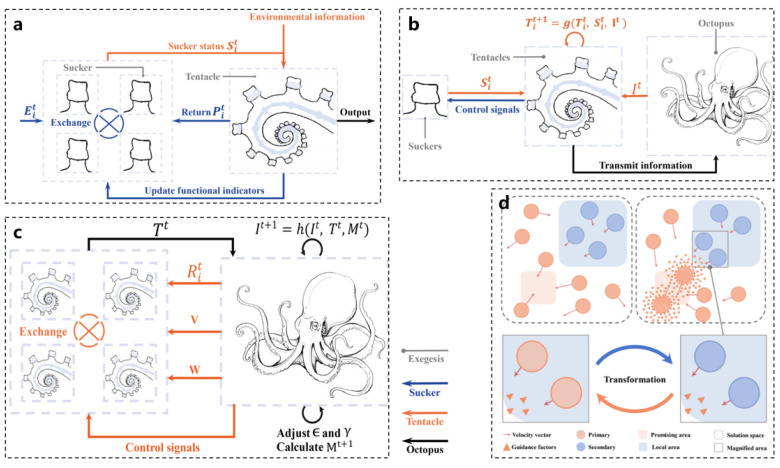
Intelligent optimization algorithms inspired by octopuses [22]. (**a**) Suction cups and perception. (**b**) Tentacles and distributed processing. (**c**) Octopuses and individual decision making. (**d**) Octopus swarms and group optimization algorithms.

The multi-level structure of the OIO consists of four levels: tentacles, suction cups, individuals, and groups, simulating the octopus’s nervous system to facilitate information transfer and interaction. As illustrated in Figure 8a, the suction cups are responsible for the perception and processing of local information. At the beginning of each iteration, information is collected through an evaluation function and relayed to the tentacles. The tentacles then adjust the parameters and update the performance indicators based on the feedback received from the suction cups. As shown in Figure 8b, the tentacles are tasked with distributed search and autonomous decision making. They integrate information from the suction cups, evaluate the environment, and adjust their movements accordingly, providing feedback to the individual and influencing subsequent movement instructions. Figure 8c depicts the role of individuals as macro decision makers, who make decisions by synthesizing information from the tentacles and environmental characteristics. They also implement a reward and punishment mechanism to adjust the group optimization algorithm, preventing prolonged stagnation in inefficient areas.

Figure 8d illustrates how the group achieves collaborative optimization by aggregating information to comprehensively describe the solution process, enabling all individuals to work in concert. The OIO utilizes an asynchronous parallel mechanism to allocate computing resources efficiently, thereby improving execution efficiency. Different tentacles and suction cups can search and process information in parallel, significantly reducing the algorithm run time. Moreover, the OIO possesses the capability for adaptive parameter control, allowing it to adjust search step sizes, exploration–exploitation balance factors, and other parameters based on real-time feedback. This adaptability enhances the robustness and convergence speed of the algorithm. A regional regeneration mechanism is employed to prevent the tentacles from remaining in local optimal areas for extended periods, thereby enhancing global search capabilities. The OIO not only emphasizes individual intelligence simulation but also highlights the emergence of group intelligence. The exchange of information between individuals improves the algorithm’s adaptability. In terms of design, the OIO rationally implements parallel and asynchronous iteration strategies, fully utilizing multi-core processor resources to avoid inefficiencies associated with synchronous updates, thus further enhancing execution efficiency. The adaptive and dynamic adjustment mechanisms enable the algorithm to modify strategies in real time according to varying optimization problems, improving its versatility. Additionally, the OIO employs various mechanisms, such as tentacle extension and contraction and regional regeneration, to avoid local optima and continuously explore new areas in search of global optimal solutions. This flexible design, combined with diverse mechanisms, equips the OIO with superior search capabilities and optimization performance when addressing complex optimization problems, offering novel ideas and methodologies for applications in related fields [22,86].

The octopus’s high-performance distributed control layer is notably distinct compared to that of other organisms. Its intricate distributed local control system enables the octopus to manage thousands of actuated degrees of freedom, specifically in its suction cups and arm musculature [80]. While it is currently unfeasible to create a robot with as many local control degrees of freedom as an octopus possesses, the architectural principles of the octopus can be effectively applied to robots with a reduced number of local control degrees of freedom.

In the era of big data, studying the nervous system of octopuses holds great significance. For example, distributed processing can allocate data across multiple nodes for parallel computing, enabling large-scale data integration. As the volume of data increases, the number of computing nodes can also be expanded, thereby enhancing the processing capacity of the entire system. In the field of deep learning, these insights have important implications for the development of edge computing, federated learning, and multimodal data processing [87,88,89]. In the future, a better understanding of octopus-distributed control could help humans develop innovative methods for robot planning and control, as well as provide new inspiration for computational optimization. Distributed processing can integrate data from various sensors in real time. Through parallel computing, perception and control tasks are distributed across multiple computing nodes, allowing robots to process complex computational tasks more quickly and enhancing the overall intelligence and flexibility of the system [90,91].

## 5. Octopus Inspires Other Aspects of Robotics

**Figure 9:**
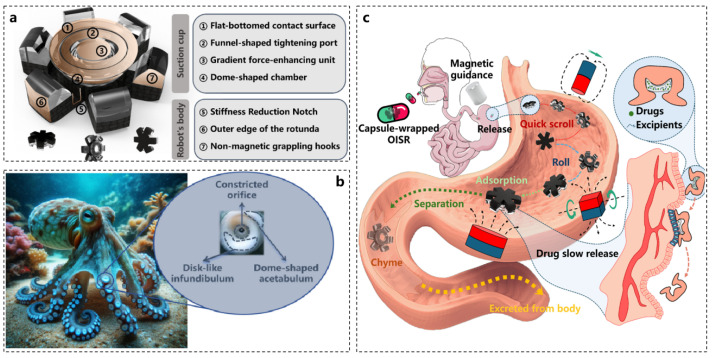
Octopus-inspired drug-releasing robot [94]. (**a**) Bionic structure. (**b**) Schematic diagram of the characteristics of the octopus suction cup. (**c**) Schematic diagram of the working of the drug-releasing robot.

In addition to inspiring the design of robotic components, octopuses also influence the development of various application fields. As shown in Figure 9b, the numerous suction cups on octopus tentacles can expel air through muscle contractions, generating a pressure difference. When the muscle tension is relaxed, the suction cups firmly adhere to objects. This phenomenon has inspired numerous research directions, including the integration of electrophysiological signal acquisition equipment with measurement targets, the development of wearable hydrogels, wound-healing methods, and high-performance robotic arm suction cups. Given the strong adhesion capabilities of octopus suction cups, incorporating this structure into the probes of electrophysiological signal acquisition equipment can ensure a secure connection to measurement targets, such as rough skin, thereby enabling the continuous monitoring of vital signs in living organisms [92,93].

Figure 9a illustrates a drug-release soft robot designed based on the structural characteristics of octopus suction cups, capable of slow drug release in the body for therapeutic purposes. The overall design of the robot is magnetic; under the influence of an external magnetic field, as depicted in Figure 9c, the soft robot can be positioned at lesion sites in the stomach and securely attach via instantaneous suction generated by the magnetic gradient force. The drug contained within the suction cup is gradually released at the lesion site, achieving targeted and sustained drug delivery [94].

**Figure 10:**
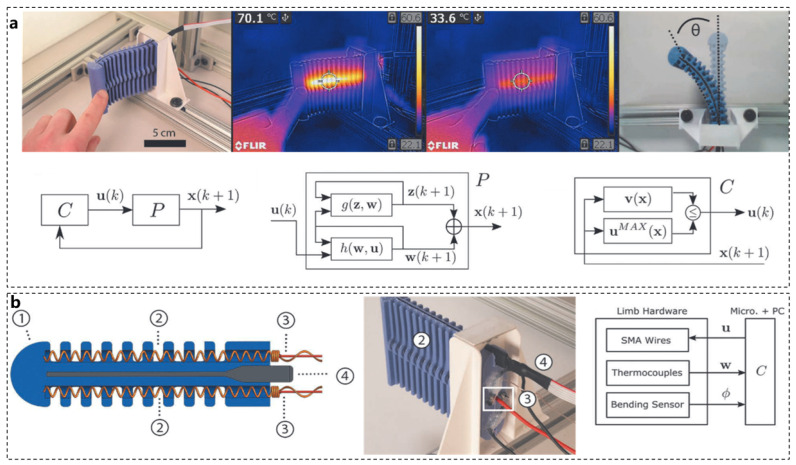
SOTIF functional design example [96]. (**a**) Test solution design and demonstration. (**b**) SOTIF functional design and system block diagram.

In addition, the development of Safety of the Intended Functionality (SOTIF) continues in the industry. The concept of SOTIF originated in the automotive industry and is defined by the ISO21448 standard [95]. It aims to address the risk of harm caused by functional limitations or reasonably foreseeable misuse under non-system failures. Its core goal is to minimize the proportion of known and unknown hazardous scenarios and improve the boundaries of the system in complex environments through design optimization and verification. We believe that this concept is also applicable to bionic robots. This paper introduces the concept of SOTIF into the study of soft robots for the first time. Research on SOTIF primarily focuses on countermeasures in the event of soft robot failures or situations outside the scope of system modeling, ensuring that the soft robot actuator does not cause harm to itself or the external environment. Similar to how an octopus protects itself in unknown situations through means such as camouflage and ink spraying, the design of predictive functional safety can guarantee the safe operation of the soft robot’s actuator during the control process. In response to these challenges, some researchers have proposed feedback strategies to ensure the safe operation of soft actuators when controlling soft robots. The supervisory controller monitors the actuator’s state and dynamically adjusts the control input to prevent conditions that could lead to physical damage. As shown in Figure 10b, relevant researchers utilized shape memory alloys as actuators along with thermal sensors. The temperature of the soft limb robot is maintained in a stable state to prevent overheating during contact scenarios, such as environmental restrictions, human interaction, or unreasonable control inputs. Figure 10a presents experimental results demonstrating that when the robot is touched, the system automatically lowers the temperature to prevent burns. The experiment confirms that the expected functional safety monitor is effective, demonstrating the stability and verifiable safety of the supervisory controller under specific conditions [96].

## 6. Conclusions

In the realm of soft robotics, the octopus serves as an excellent teacher. From it, we can derive insights into sensor design and fusion, innovative actuator design, optimization of processing systems, and application ideas across various fields. This paper discusses the application of advanced materials inspired by octopuses in the design of soft robot sensors, including new combinations of classic sensor materials to achieve novel sensor functions and improved sensor performance driven by octopus designs.

In the area of actuator design, we have presented the latest research progress from perspectives including pneumatic, hydraulic, electric, and energy-derived actuation, along with the development of new actuator materials, drive structures, and control algorithms that enhance control accuracy, inspired by the movement and locomotion of octopuses.

Regarding processing systems, we explored the design and optimization of distributed systems inspired by octopuses and introduced intelligent optimization algorithms based on octopus behavior. Finally, we integrated the concept of expected functional safety into soft robot design and outlined its practical applications.

Human exploration of the ocean is deepening, biotechnology is advancing rapidly, and research on mollusks, such as octopuses, is becoming increasingly sophisticated. Through bionic research on octopuses, combined with the continuous progress in artificial intelligence and materials science, breakthrough developments are expected in many fields [4,97]. The tentacles of octopuses exhibit exceptional freedom of movement and flexibility, while their unique nervous system and learning capabilities provide a rich source of inspiration for artificial intelligence and robotics. In the realm of intelligent soft robots, future bionic octopus robots will be able to simulate octopus movements more accurately, optimize grasping paths and strengths using artificial intelligence algorithms, and enhance their adaptability in complex environments [98,99].

In the realms of human–computer interaction and medical applications, octopus-like flexible robotic arms are expected to see widespread use in surgical procedures and rehabilitation treatments [17,76,100]. Their softness and high degree of freedom enable more precise operation of surgical instruments, thereby reducing trauma to patients. Additionally, when combined with artificial intelligence and flexible materials, smart wearable devices resembling octopus tentacles may be developed in the future for rehabilitation training or assisted movement [101].

In the context of integrating artificial intelligence and neuroscience, the nervous system and learning capabilities of the octopus offer a new research direction for artificial intelligence. By simulating the octopus’s neural network, more efficient decision-making algorithms can be developed in the future, enabling robots to autonomously learn and adapt to complex environments [22,102,103,104,105,106,107,108]. Additionally, further research into the learning and memory mechanisms of octopuses may lead to breakthroughs in artificial intelligence within the fields of cognitive and emotional computing. Regarding multifunctional integrated bionic systems, robots that can monitor environmental changes in real time and autonomously adjust their behavior will be developed by combining the octopus’s perceptual abilities with the responsiveness of smart materials [32,101,102,103,104,105,106].

## Figures and Tables

**Table 1:** Comparison of the advantages and disadvantages of Kalman filters, CNNs, and RNNs in the sensor fusion of soft robots.

Method	Advantages	Disadvantages	Applicable Scenarios
Kalman	Real-time performance Efficient calculation Multidimensional data fusion	Dependent linear models Noise sensitivity	Soft tentacle pose estimation Dynamic environment position
CNN	Spatial feature extraction High-dimensional data processing End-to-end learning	Highly data-dependent Limited real-time performance	Object recognition (grasping) Tactile texture perception
RNN/LSTM	Temporal modeling capabilities Dynamic adaptation Multi-modal fusion	High training complexity Computational delay	Continuous motion planning Abnormal state detection

**Table 2:** Comparison between octopus-inspired soft robot actuators and other biomimetic soft robot actuators.

Bionic Objects	Design Features	Drive Mode	Application Scenarios	Technical Challenges and Limitations
Octopus	(1) Highly flexible (2) Dynamic grasping (3) Stable attachment	Pneumatic/hydraulic drive SMA	Flexible gripping Underwater detection Medical endoscope	Nonlinear deformation control is complex
Elephant Trunk	(1) Flexible and rigid, can carry heavy objects (2) Layered muscle structure	Pneumatic/hydraulic drive	Industrial handling Rescue robots	Insufficient dynamic stability under high loads
Earthworm	(1) Segmented structure (2) Low energy consumption	Pneumatic Electroactive polymers	Underground exploration Pipeline inspection	Slower movement speed
Fish	(1) Streamlined body (2) Low noise	Motor Electroactive polymers	Marine ecological monitoring Underwater military reconnaissance	Limited steering flexibility

## Data Availability

Not applicable.
